# Burying Alive

**Published:** 1859-10

**Authors:** 


### BURYING ALIVE.

"'Tis well," were the last recorded words of the great Washington, uttered in reference to his burial.

"Do not let my body be put into the vault in less than three days after I am dead," and, looking earnestly into his secretary's face he continued, "Do you understand me?" "Yes," said Mr. Lear. "'Tis well," replied Washington, and spoke no more.

The great Dr. Physic left an injunction that a blood vessel should be severed before he was buried, in order to make it certain that he was dead.

The marvellous stories put in circulation by the credulous, in reference to the turning of bodies, and the tearing of the grave clothes in the fearful struggle for breath, are without any rational foundation. If a hot iron raises no blister on the skin, or if a severed artery does not bleed, there can be no reasonable ground for doubting that death has taken place. These tests should be applied not sooner than eight or ten hours after the apparent decease.

